# Rare double orifice mitral valve malformation associated with bicuspid aortic valve in Turner syndrome: diagnosed by a series of novel three-dimensional echocardiography and literature review

**DOI:** 10.1186/s12872-021-02184-2

**Published:** 2021-08-04

**Authors:** Feifei Sun, Xueying Tan, Aijiao Sun, Xintong Zhang, Yanxiao Liang, Weidong Ren

**Affiliations:** 1 Department of Ultrasound, Shengjing Hospital of China Medical University, No. 36 Sanhao Street, Heping District, Shenyang, 110004 China; 2 Department of Cardiac Surgery, Shengjing Hospital of China Medical University, Shenyang, China

**Keywords:** Double orifice mitral valve, Bicuspid aortic valve, Three-dimensional echocardiography, Congenital cardiac malformation, Turner syndrome, TrueVue, TrueVue Glass, TouchVue

## Abstract

**Background:**

Patients with both double orifice mitral valve (DOMV) and bicuspid aortic valve (BAV) malformation are rare. Although DOMV or BAV can be detected in some genetic syndromes, it has not been reported to simultaneously appear in Turner syndrome (TS). TrueVue, TouchVue, and TrueVue Glass are the latest technologies in advanced three-dimensional echocardiography (3DE), which is an important information supplement to two-dimensional echocardiography (2DE) for the diagnosis of congenital cardiac malformations. Herein we report the novel use of the above-mentioned technologies in the diagnosis and evaluation of a rare, combined valve malformation. Meanwhile, we also reviewed the literature for cases involving both DOMV and BAV and their association with various genetic syndromes.

**Case presentation:**

We present the case of a 5-year-old girl diagnosed with TS because of a developmental delay. DOMV and BAV were found through echocardiographic examination. Three-dimensional transthoracic echocardiography as well as a series of novel advanced techniques were applied to clearly display the spatial structure of all tiers of the mitral valve apparatus, aortic valve, and arch to facilitate an accurate diagnosis.

**Conclusions:**

This is the first case in which both DOMV and BAV were associated with TS. Innovative TrueVue and TrueVue Glass offer unprecedented photographic stereoscopic images, while TouchVue technology greatly improved the ultrasonic diagnostic workflow and the diagnostic performance of rare valve malformations by adding virtual light sources to display realistic light-shadow effects.

**Supplementary Information:**

The online version contains supplementary material available at 10.1186/s12872-021-02184-2.

## Background

The double orifice mitral valve (DOMV) is considered a rare congenital heart malformation due to the abnormal embryonic development of the endocardial cushion and myocardium, characterized by a two-channel atrioventricular valve in the left ventricle and accounting for about 0.05% of all congenital heart diseases [1, 2]. The two orifices of the mitral valve (MV) can be symmetrical or asymmetrical, isolated or exist with other cardiac malformations, and can be divided into complete bride type, incomplete bridge type, or hole type [3, 4]. A bicuspid aortic valve (BAV) is relatively common and is characterized by the abnormal fusion of two leaflets of the aortic valve (AV) during development, resulting in a two leaflet valve instead of the normal tricuspid AV [5]. Patients with both DOMV and BAV are rare [6].

Turner syndrome (TS), known as congenital chromosomal disorder, is caused by the partial or complete deficiency of the X chromosome and is reported to have a higher incidence of congenital heart diseases, of which AV malformation is one of the most common changes [7, 8]. Reported mitral malformations in TS are MV prolapse, myxomatous degeneration, parachute-like MV, cleft, and accessory leaflet [9–12]. To our knowledge, the occurrence of DOMV in TS has not been reported.

The two-dimensional echocardiography (2DE) is the most common and important method for detecting congenital heart valvular disease [13]. Meanwhile, the role of real-time three-dimensional echocardiography (RT-3DE) in the diagnosis and evaluation of congenital heart diseases has been receiving more attention due to its ability to show the overall spatial structure of the heart [14]. Several novel 3DE imaging technologies have been developed by Philips Medical System recently. TrueVue technology, which came out in 2019, is a new, high-resolution 3D echocardiographic imaging mode that can clearly display the subtle structure of the heart, which makes the ultrasound image more closely resemble the real anatomical pathology [15]. The TrueVue Glass, released globally in 2020, displays the myocardial tissue outside the heart cavity in a transparent mode, focusing on the cardiac cavity filled with blood flow, and can display the thin and translucent valves, which provides both a practical value and technological sense during the diagnosis and evaluation of heart diseases [16].

Here, we report a case with TS and congenital cardiac valvular malformations with DOMV and BAV, wherein we applied these advanced 3DE imaging technologies, which played an important role by providing images and diagnostic information that were previously unavailable.

## Case presentation

A 5-year-old girl presented to our hospital with a complaint of short stature and mild backache. She was found to have vitamin D deficiency, spina bifida, and small pituitary volume. Her peripheral blood chromosome result indicated “45, X,” consistent with TS.

**Fig. 1:**
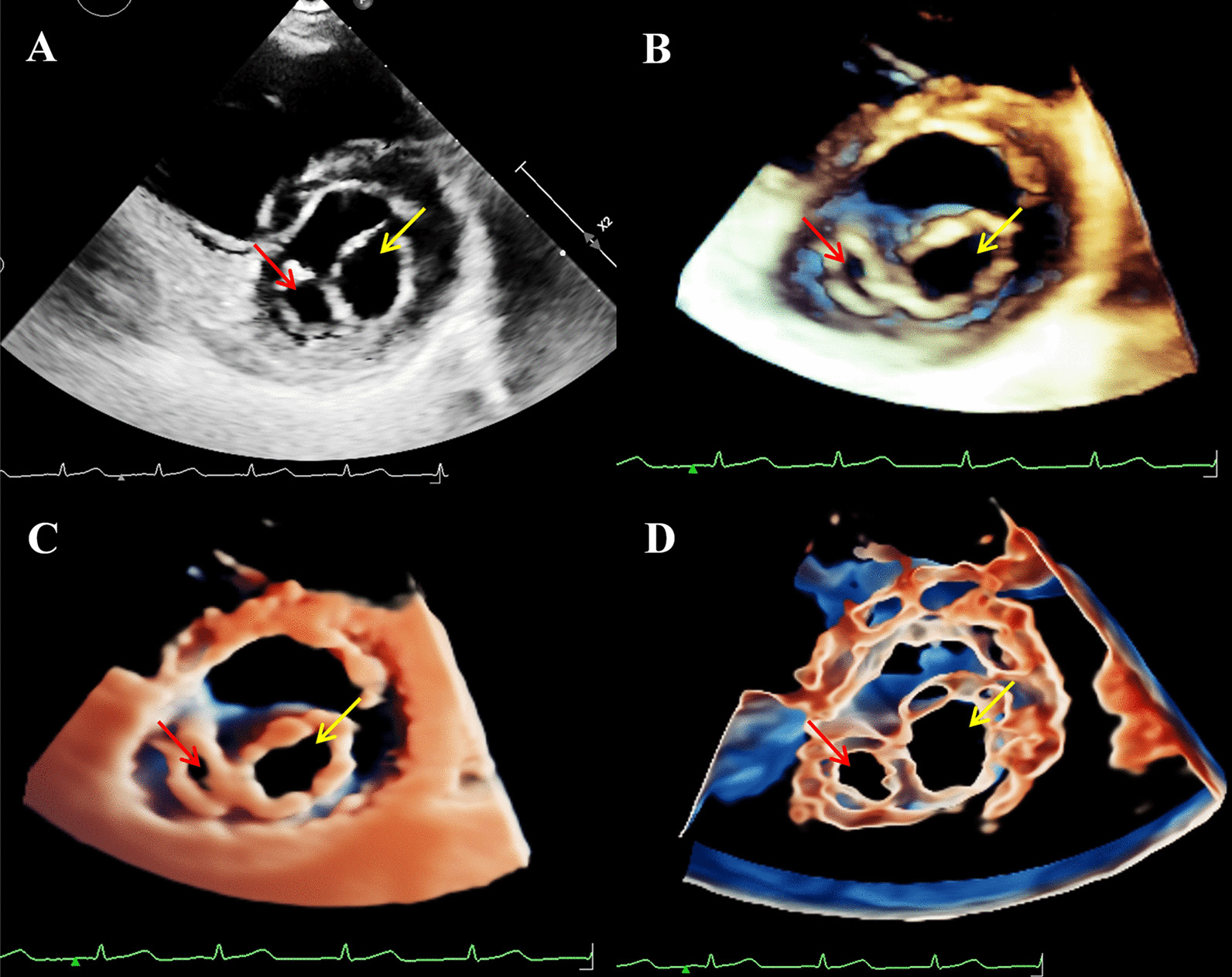
Double mitral orifices. **A**–**D** 2D-TTE, traditional 3DE, TrueVue, and TrueVue Glass images showing double mitral orifices viewed in left ventricular short axis section (mitral valve orifice level). Yellow arrows show orifice 1, red arrows show orifice 2. *2D-TTE* two-dimensional transthoracic echocardiogram, *3DE* three-dimensional echocardiography

**Fig. 2:**
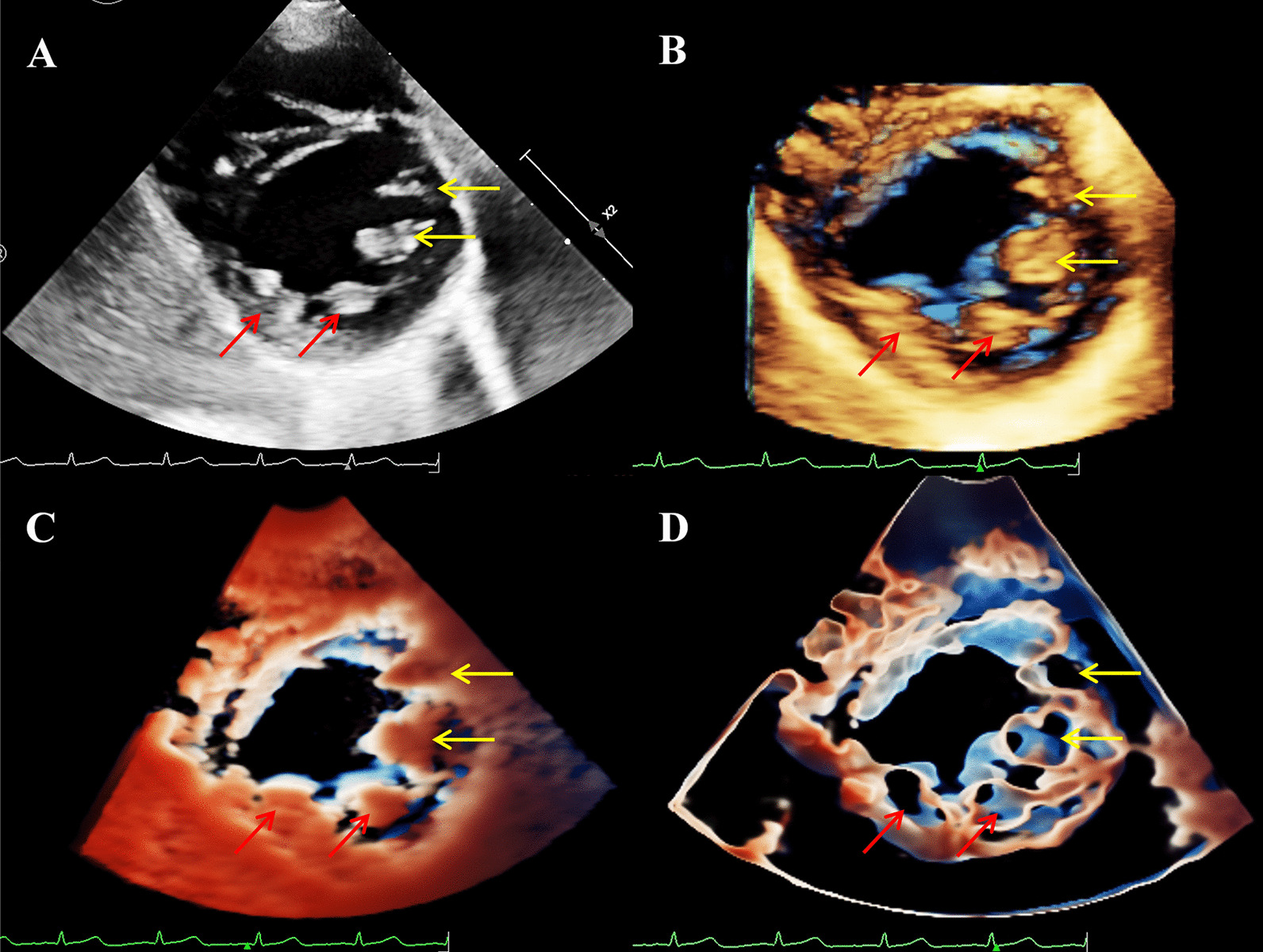
Papillary muscles. **A**–**D** 2D-TTE, traditional 3DE, TrueVue, and TrueVue Glass images showing the four papillary muscles in the left ventricular short axis section (papillary muscle level). Yellow arrows show the papillary muscles of orifice 1, red arrows show the papillary muscles of orifice 2 of the mitral valve. *2D-TTE* two-dimensional transthoracic echocardiogram, *3DE* three-dimensional echocardiography

**Fig. 3:**
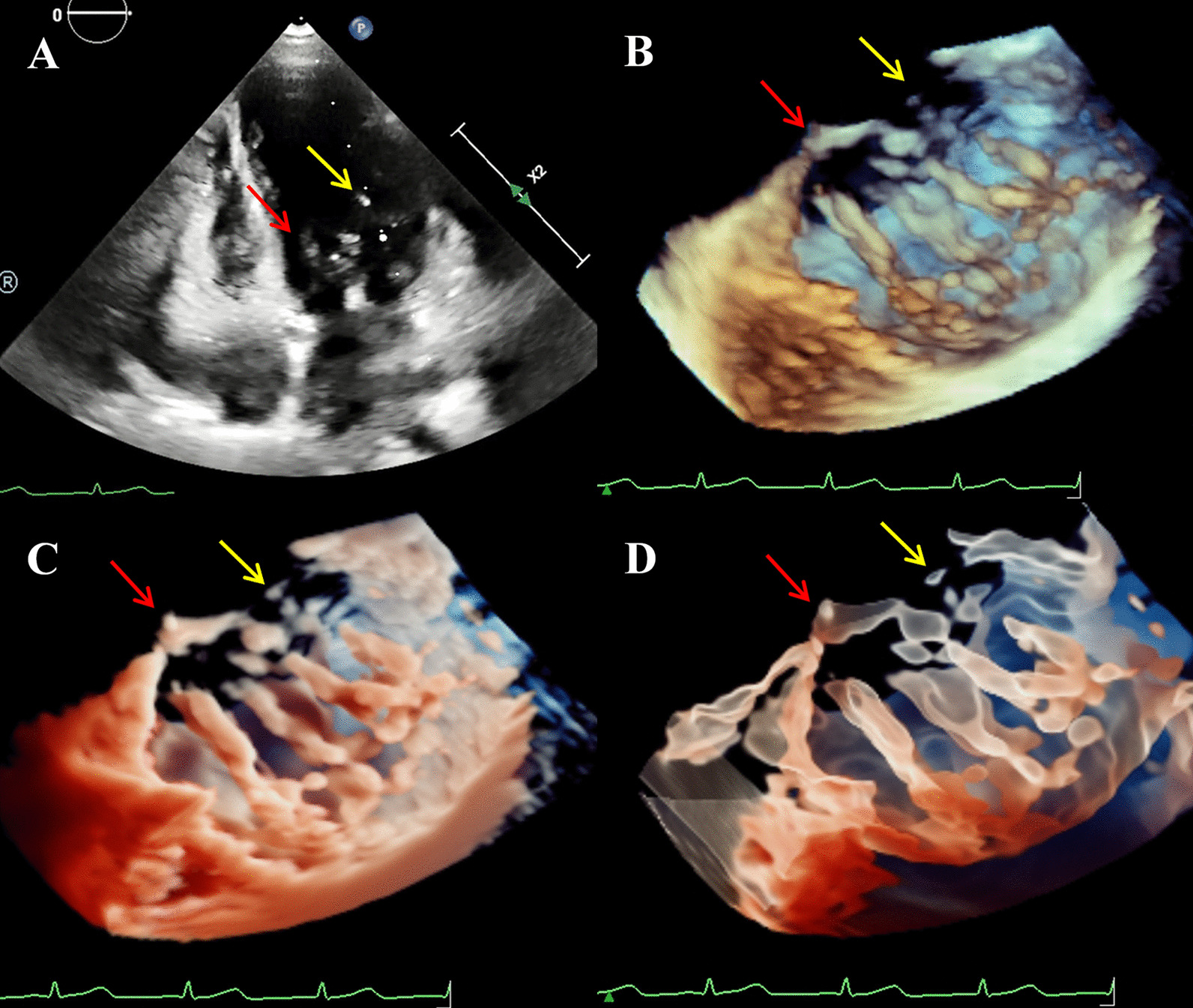
Chordae tendineae. **A** 2D-TTE showing the long axis of the chordae tendineae. **B**–**D** demonstrate the spatial morphological characteristics of the chordae tendineae connecting the two orifices with the four papillary muscles of the mitral valve apparatus by traditional 3DE, TrueVue, and TrueVue Glass. Yellow arrows indicate chordae of orifice 1, red arrows indicate chordae of orifice 2. *2D-TTE* two-dimensional transthoracic echocardiogram, *3DE* three-dimensional echocardiography

**Fig. 4:**
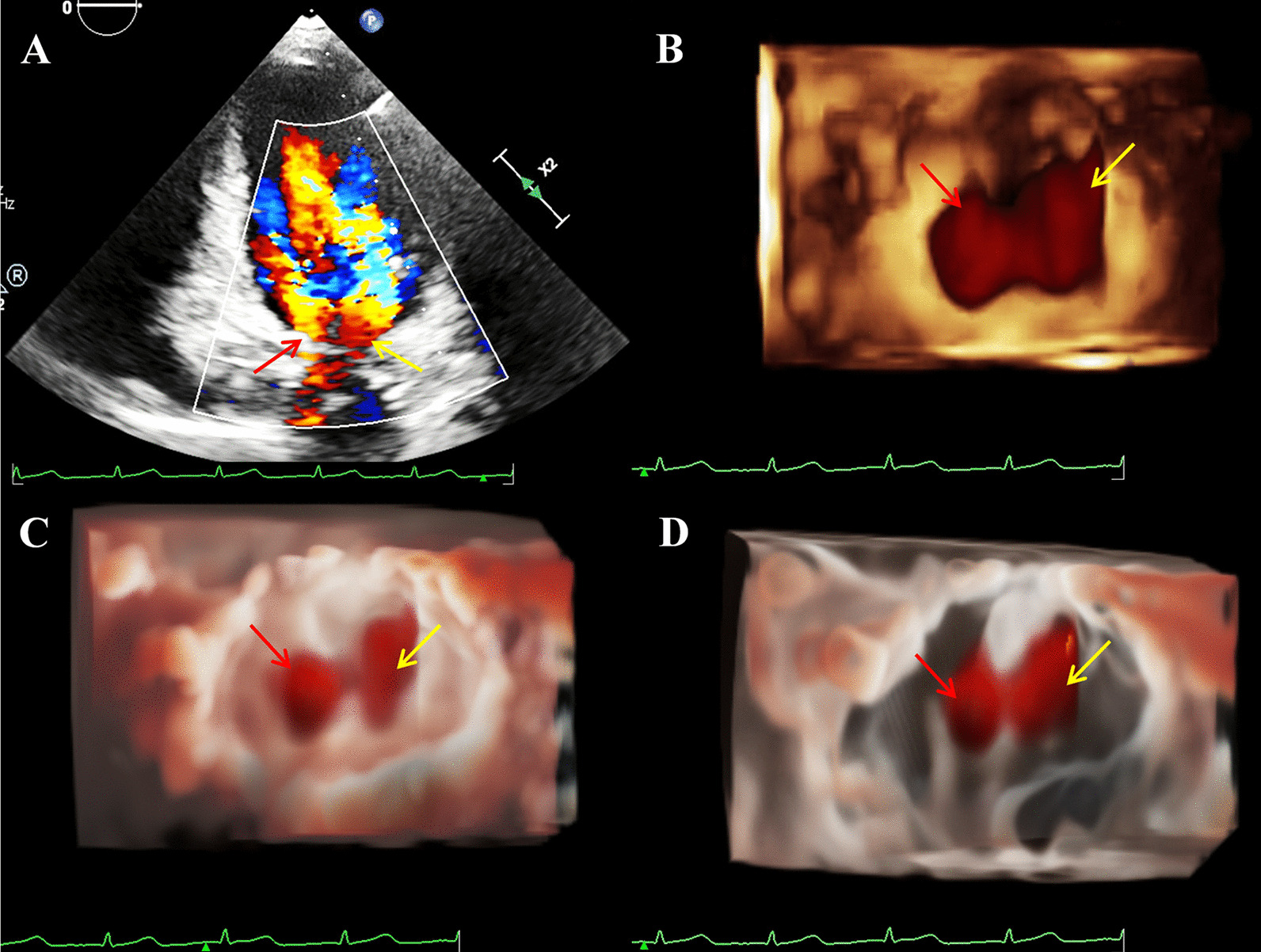
Two separate diastolic mitral inflow flows. **A** 2D-TTE color Doppler in the non-standard apical 4-chamber view shows that there are two blood streams flowing through the left atrioventricular channel simultaneously. **B**–**D** Traditional 3DE, TrueVue, and TrueVue Glass images showing two jets into the left ventricle. Yellow arrows show jets from orifice 1, red arrows show jets from orifice 2. *2D-TTE* two-dimensional transthoracic echocardiogram, *3DE* three-dimensional echocardiography

**Fig. 5:**
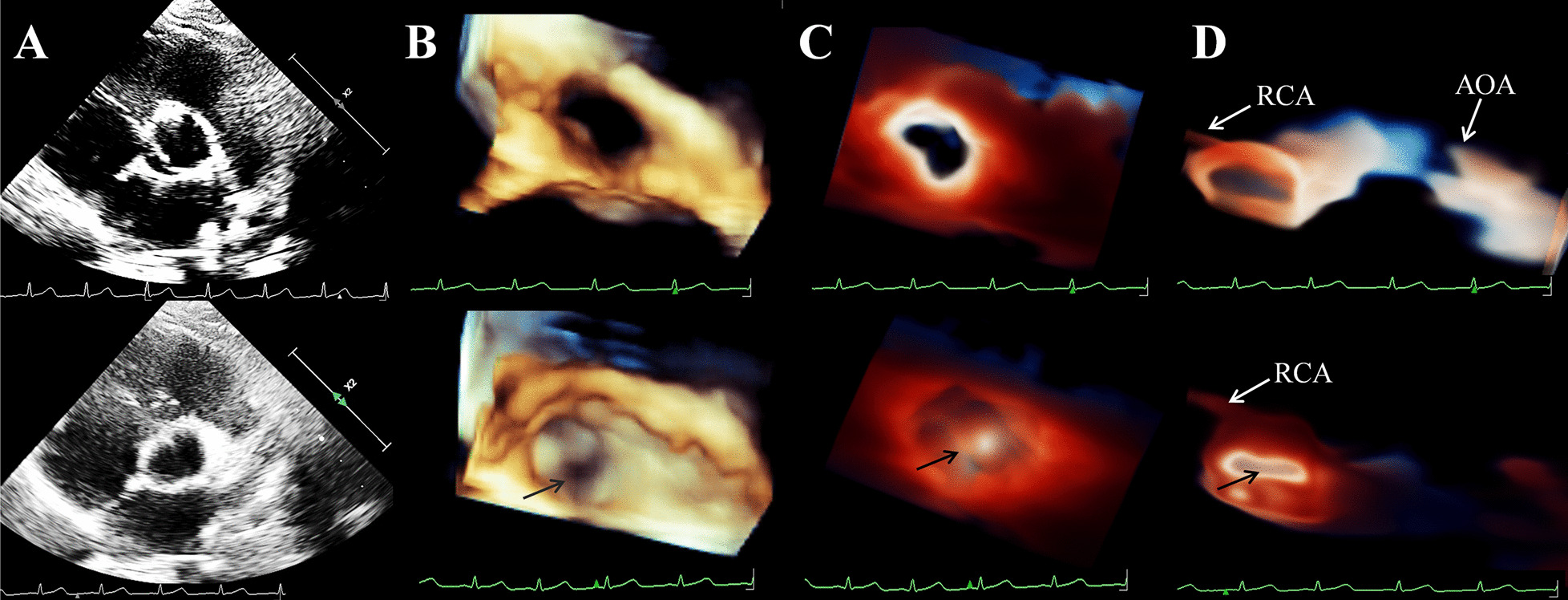
Bicuspid aortic valve. **A** 2D-TTE showing the aortic valve during systole (above) and diastole (below). **B**–**D** Traditional 3DE, TrueVue, and TrueVue Glass images demonstrating aortic valves when opened and closed, respectively. Black arrows show aortic valve closing line. *AOA* aortic arch, *RCA* right coronary artery, *2D-TTE* two-dimensional transthoracic echocardiogram, *3DE* three-dimensional echocardiography

To rule out developmental delay due to heart disease or cardiac abnormalities associated with TS, the transthoracic Echocardiogram (TTE) was performed using Philips EPIQ CVx cardiovascular specific ultrasonic diagnostic equipment (Philips Medical System, Andover, MA, USA). The 2DE scan application was conducted via a S9-2 (2–9 MHz) probe. The X5-1 (1–5 MHz) probe was used for the 3DE examination. As diagnoses of both DOMV and BAV were suspected, the MV apparatus and AV-related structures were examined by 3D-TTE for further morphological and functional evaluation. On the basis of traditional 3D imaging, we launched the new TrueVue imaging mode. In TouchVue, in addition to being able to directly zoom and rotate the image with two fingers on the touch screen, more importantly, a single finger click on the target structure can display and move the position and depth of a virtual light source. Based on the TrueVue image, we further applied the Glass mode to obtain a transparent rendering effect. The short-axis view at the level of the MV via above-mentioned four techniques demonstrated that there were asymmetric double left atrioventricular orifices divided by a complete fibrous bridge, of which the anterolateral orifice was larger (Fig. 1). The total area of the two mitral orifices was about 2.17 cm^2^. At the level of the papillary muscle, four papillary muscles were revealed by 2D-TTE, traditional 3D-TTE, and TrueVue images, but were transparent and only the borders were revealed in the TrueVue Glass image (Fig. 2). The spatial relationship demonstrated that the two orifices were connected by their respective chordae tendineae, separating the two papillary muscles of the same side (Fig. 3). Color Doppler was superimposed, demonstrating two jets through the respective channels of the two orifices (Fig. 4). The real-time dynamic TrueVue Glass image can visualize the 3D blood flow through the thin and transparent valve (Additional file 1: Video 1). In this patient with MV malformation, we initiated the “dual volume” mode so that we could simultaneously observe two orifices on the left atrial view, four papillary muscles on the left ventricle view and the flow in two opposite directions. (Additional file 2: Video 2). There was a slight increase in the forward blood flow velocity of the left atrioventricular valve orifices, the velocity in the early diastole was about 1.2 m/s, and in the late diastole was about 0.7 m/s. In addition, the short-axis view of the heart base revealed a BAV, with two leaflets in the left anterior and right posterior directions. We simultaneously compared the two-dimensional, traditional 3D, and new 3D TrueVue combined with the light and Glass mode imaging. Via TrueVue Glass imaging, when the light source was placed behind the valves, we observed a straight-line AV closure shape; the emergence of the coronary arteries and the spatial character of the aortic arch were also displayed simultaneously (Fig. 5). There was no aortic stenosis (peak systolic velocity is 1.3 m/s) or regurgitation, and the aortic arch descended to the left normally. Trivial mitral regurgitation was observed, but no other associated congenital cardiac abnormalities were detected. Surgical intervention was not performed for the time being because there was no significant regurgitation or stenosis. The results of the echocardiographic follow-up after three months, half a year, and 1 year showed that there was no obvious stenosis or insufficiency of the valves.

## Discussion and conclusions

We further reviewed 24 patients with both DOMV and BAV in 16 references from 1988 to 2020 (Table 1). These patients ranged in age from one month to 67 years old (25.55 ± 19.95), including 12 (50.0%) adults, 10 (41.7%) children or adolescents, and 2 (8.3%) patients whose ages were not described. Overall, 15 (62.5%) were male, 7 (29.2%) were female, and 2 (8.3%) were of undescribed gender. Except for 1 (4.2%) case in which the specific DOMV type was unknown, all the other cases were of complete bridge type, and in patients with relevant record information, there was an even distribution of symmetrical and asymmetrical orifices. As for the dysfunction of the MV and AV, mitral regurgitation was the most common, present in 10 (41.7%) patients, aortic stenosis in 8 (33.3%), mitral stenosis in 7 (29.2%), and aortic regurgitation in 4 (16.7%). Chordae tendineae was noted in 12 (50.0%) patients: the chordae attached to each orifice in 5 (20.8%); accessory septal attachment in 2 (8.3%); parachute chordal attachments in 2 (8.3%); and attachment to the anterior wall, chordal ring, non-elongation, and one chorda attachment to the bridging structure were seen in 1 (4.2%) case each. Papillary muscles were mentioned in 14 cases (58.3%). They were normal in 6 (25.0%) and fused in 3 (12.5%); the morphology was abnormally displaced and unequal in a normal number of papillary muscles in 2 (8.3%), and four or more papillary muscles were noted in 3 (12.5%). The most complicated malformation was coarctation of the aorta (n = 11, 45.8%). All patients underwent echocardiography, and among them, 10 (41.7%) underwent transesophageal echocardiography (TEE) and 9 (37.5%) underwent 3DE. Of the 13 (54.2%) patients with operation-related information, 8 (33.3%) underwent operations, although some of them underwent an operation to correct the aortic constriction rather than repair or replace the deformed valve. None of the reported cases involved a genetic syndrome except our case.

**Table 1:** Summary of the literature about patients diagnosed with DOMV and BAV

No.	Study	Age (years)	Gender	Clinical manifestation	DOMV type	Function of MV and AV	MV chordae tendineae	MV papillary muscles	Associated heart diseases (except for BAV)	Diagnose methods	Surgery	Genetic
1	Baño-Rodrigo et al. [26]	–	–	–	CB	–	–	Fused	Mitral valve cleft, common atrium, CoA, PDA, hypoplastic left heart syndrome	2D-TTE, postmortem	–	–
2	As above	–	–	–	–	–	–	–	–	As above	–	–
3	Gerber et al. [27]	63	M	Heart failure	CB, Sym	Severe MR	–	–	Dilated cardiomyopathy	2D-TTE	Y	–
4	Karas et al. [28]	23	F	Asymptomatic	CB, Sym	Mild AS	–	At least 4	N	2D-TTE, 2D-TEE	N	–
5	Proença et al. [29]	32	M	Hypertension	CB, Sym	Major AR	–	–	CoA, aneurysm of Valsalva sinus	2D-TTE, 2D-TEE, CMR	Y	–
6	Das et al. [1]	0.08	M	–	CB, Sym	Moderate MS, mild MR	Accessory septal attachment	Fused	CoA, subaortic stenosis, dysplastic TV	2D-TTE	–	–
7	As above	1	F	–	CB, Asy	Mild MS, AS	Accessory septal attachment	2 displaced	CoA	As above	–	–
8	As above	7	F	–	CB, Sym	Normal	U	Normal	CoA	As above	–	–
9	As above	9	M	–	CB, Asy	Mild MS, AS	U	Fused	VSD	As above	–	–
10	As above	12	F	–	CB, Sym	Mild MR, AR	Attachment to anterior wall	Normal	N	As above	–	–
11	As above	12	M	–	CB, Asy	Normal	Chordal ring, parachute chordal attachments	2 unequal	CoA, VSD	As above	–	–
12	Erkol et al. [30]	32	M	Hypertension, shortness of breath	CB, Sym	Moderate AS, mild AR	Parachute chordal attachments	Multiple	N	2D-TTE, 2D-TEE, CMR	–	–
13	Aggarwal et al. [31]	35	F	Exertional chest tightness	CB, Asy	Sever AS, mild AR, trivial MR	U	Normal	CoA	2D-TTE, 2D-TEE, 3D-TEE	–	–
14	Lee et al. [32]	41	M	Palpitation	CB, Sym	Moderate MS	One chorda attachment to bridging structure	–	N	2D-TTE, 3D-TEE	–	–
15	Kharwar et al. [33]	15	M	Dyspnea on exertion,	CB, Asy	Mild MS, moderate MR	U	Normal	N	2D-TTE, 3D-TTE	Y	–
16	Kocabaş et al. [14]	16	M	–	CB, Sym	Mild MR	–	–	CoA	2D-TTE, 3D-TTE	N	–
17	Mouine et al. [34]	15	M	Hypertension	CB, Asy	Normal	–	–	CoA, VSD	2D-TTE	Y	–
18	Saylik et al. [35]	21	M	Exercise-induced chest pain, palpitations	CB, Asy	Severe MS	–	–	N	2D-TTE, 2D-TEE	Y	–
19	Khani and Rohani [36]	54	F	Dyspnea on exertion	CB, Asy	Moderate AS	–	–	N	2D-TEE, 3D-TEE	N	–
20	Yang et al. [37]	57	M	Hypertension	CB, Sym	Mild AS, trivial MR	–	–	CoA, aortic aneurysm	2D-TTE, 3D-TEE, CMR	Y	–
21	Benjamin et al. [3]	67	M	Short of breath	CB, Asy	MR, severe AS	–	Normal	Pulmonary vein stenosis	2D-TTE, 3D-TEE, CTA	Y	–
22	Bayat et al. [6]	25	M	Hypertension, undetectable distal pulses	CB, Sym	Trivial MR	–	–	CoA, aberrant left subclavian artery	2D-TTE, 3D-TTE, TEE, CTA	Y	–
23	Fernandez Gasso et al. [24]	20	M	Asymptomatic	CB, Asy	Mild to moderate MR	Non-elongation	Normal	Myxomatous multivalvular disease, TV prolapse, PV dysplasia	2D-TTE	N	–
24	This case	5	F	Short stature, mild backache	CB, Asy	Mild MS	U	4	N	2D-TTE, Novel 3D-TTE	N	TS

– not mentioned, *Asy* asymmetric, *CB* Complete bridge type, *CoA* coarctation of the aorta, *CMR* cardiac magnetic resonance imaging, *CTA* cardiac computed tomographic angiography, *MR* mitral regurgitation, *MS* mitral stenosis, *MV* mitral valve, *N* no, *PV* pulmonary valve, *Sym* symmetric, *TS* turner syndrome, *TTE* transthoracic echocardiogram, *TV* tricuspid valve, *U* chordae attach to each orifice respectively, *VSD* ventricular septal defect, *Y* yes

An invasive TEE is not usually necessary for diagnosis due to the excellent acoustic window. Clear display of children’s valves via traditional 2DE and 3DE has certain difficulties due to their thinness, easily leading to misdiagnosis by inexperienced sonographers [17]. TrueVue is a novel 3D rendering mode with high resolution [18]. By changing the position and depth of the light source, realistic light and shadow effects can be obtained while illuminating the target structure, simulating it, including the texture of the double orifices and the sub-valvular apparatus, into photorealistic 3D images. TouchVue provides an operating platform that can effectively improve work efficiency. The TrueVue Glass imaging shields the myocardial tissue, specifically the blood-containing heart chambers and blood vessels, depicting thin BAV in its entirety. Especially when the light source was strategically placed behind the valve, light transmitted from the opening valve clearly revealed that the “fish mouth” bicuspid valve was completely different from the “inverted triangle” of the three-leaflet AV. Even the contour of the aortic arch and right coronary artery lumens were displayed clearly in TrueVue Glass images when viewing the short axis of the AV, which cannot be seen in traditional 3D or TrueVue images. Color on all three rendering modes showed two exact jets from the left atrium side, confirming the diagnosis of 2D-TTE. The main advantages and disadvantages of these imaging methods developed by Philips company in the diagnosis of this case are summarized in Table 2. Another limitation of these new series of 3D ultrasound techniques is that they require an uncomplicated training of methods and techniques before they can be used effectively. To our knowledge, this is the first case diagnosed with DOMV and BAV using the above series of novel 3DE techniques.

**Table 2:** Comparison of the advantages and disadvantage of 2DE, traditional 3DE, TrueVue, and TrueVue Glass

	2DE	Traditional 3DE	TrueVue	TrueVue Glass
Advantages	Provide sectional morphology images	Display 3D geometry of structures	Advantages of traditional 3DE	Shield the myocardial tissue
	High resolution	Show spatial position of MV apparatus	Higher resolution	Depict edges of orifices
		Delineate anomalous attachments of the sub-valvular apparatus	Realistic light and shadow effects	Show overall aortic valve, arch and coronary artery in its entity
			Simulate the pathological texture of valves and myocardium	Simulate the thin and translucent leaflets
				Show global 3D hemodynamic characteristics
Disadvantages	Cannot see the spatial structure characteristics	The texture of the lesion quite different from the real pathological specimen	Shows that thin valves and chordae may appear false echo loss	The scraggly fine texture on the surface of the anatomical structure is insufficient to display
	Multiple ultrasound views are needed to clarify the anatomy of each component of the Mitral valve apparatus	The level of structure is unclear	Cannot see the surrounding anatomy through the valve	
	The lesion is not intuitive, the diagnosis depends more on the experience of the imaging doctor	The boundary is not clear enough		
	Time-consuming and laborious			

*2DE* two-dimensional echocardiography, *3DE* three-dimensional echocardiography, *AV* aortic valve, *MV* mitral valves

Lesions of the MV apparatus sometimes occur simultaneously and do not involve just the leaflets [19]. In this case, DOMV was first detected, and then the abnormal number of papillary muscles was noted. BAV can be associated with specific syndromes, like Marfan, William Beuren, Andersen, Bosley-Salih-Alorainy, Athabascan Brainstem Dysgenesis, Turner, and Vascular Ehlers-Danlos syndromes [5]. Furthermore, three DOMV cases with hereditary syndromes (Ellis-van Creveld, Sotos, and Holt–Oram syndrome) have been reported [20–22]. In addition, the fact that BAV occurs more frequently in males and in patients with TS suggests a potential X-linked inheritance [7, 23]. Fernandez Gasso L presented an idea that multivalvular disease may be genetic [24]. Based on these findings, our case may be a hint that multivalvular disease is associated with the X chromosome.

The patient is not currently undergoing surgery because there is no significant valve regurgitation or stenosis. However, with aging, valve elasticity decreases, which will aggravate valve dysfunction [25]; therefore, long-term follow-up of this patient is essential.

In conclusion, this case extended the possible complex lesions in TS and endorses 3D transillumination rendering techniques in detecting valvular malformations.

## Supplementary Information

**Additional file 1: Video 1.** TrueVue Glass color Doppler shows the two streams of blood entering the left ventricle from the left atrium through the double orifice mitral valve, viewed from the side of the left ventricle.

**Additional file 2: Video 2.** The short-axis view of the left ventricle shows the two orifices of the mitral valve and the corresponding four papillary muscles simultaneously through the 3D “dual volume” imaging mode.

## Data Availability

All data generated or analyzed during this study are included in this published article.

## Abbreviations

2DE: Two-dimensional echocardiography
3DE: Three-dimensional echocardiography
BAV: Bicuspid aortic valve
DOMV: Double-perforated mitral valve
MV: Mitral valve
TS: Turner syndrome
TEE: Transesophageal echocardiography
TTE: Transthoracic echocardiography
